# VEGF Is Involved in the Increase of Dermal Microvascular Permeability Induced by Tryptase

**DOI:** 10.5402/2012/941465

**Published:** 2012-05-15

**Authors:** Qianming Bai, Xiaobo Li, Xinhong Wang, Yali Xu, Li Wang, Qingyong Zhang, Lianhua Yin

**Affiliations:** ^1^Department of Physiology and Pathophysiology, Fudan University Shanghai Medical College, Shanghai 200032, China; ^2^Department of Cardiology, Shanghai Jiaotong University Affiliated Sixth People's Hospital, Shanghai 200032, China

## Abstract

Tryptases are predominantly mast cell-specific serine proteases with pleiotropic biological activities and play a critical role in skin allergic reactions, which are manifested with rapid edema and increases of vascular permeability. The exact mechanisms of mast cell tryptase promoting vascular permeability, however, are unclear and, therefore, we investigated the effect and mechanism of tryptase or human mast cells (HMC-1) supernatant on the permeability of human dermal microvascular endothelial cells (HDMECs). Both tryptase and HMC-1 supernatant increased permeability of HDMECs significantly, which was resisted by tryptase inhibitor APC366 and partially reversed by anti-VEGF antibody and SU5614 (catalytic inhibitor of VEGFR). Furthermore, addition of tryptase to HDMECs caused a significant increase of mRNA and protein levels of VEGF and its receptors (Flt-1 and Flk-1) by Real-time RT-PCR and Western blot, respectively. These results strongly suggest an important role of VEGF on the permeability enhancement induced by tryptase, which may lead to novel means of controlling allergic reaction in skin.

## 1. Introduction

Mast cells are critical for allergic inflammatory responses and cutaneous hypersensitivity reactions, such as atopic dermatitis, contact dermatitis, eczema and nettle rash [1–4]. Mast cells can be activated to release a diverse array of potent biologically active products and cytokines [5–7]. The major secretory product of human mast cells is the serine proteinase tryptase (tetrameric trypsin-like substrate specificities), which is emerging as a major mediator of allergic disease and as a promising target for therapeutic intervention [8]. Human mast cells contain at least two tryptases, *α*-tryptase and *β*-tryptase. Human mature *β*-tryptase is stored in the mast cells granules and released upon activation while *α*-tryptase is apparently processed only to the proenzyme stage and is constitutively secreted along with *β* protryptase [8, 9]. In healthy individuals, only *α*-tryptase can be detected whereas *β*-tryptase is undetectable. However, significant elevations of circulating *β*-tryptase levels were observed in patients with allergic diseases [10, 11].

The common clinical sign of allergic hypersensitivity reactions in skin is edema, which caused by increases in vascular permeability [12, 13]. It is reported that tryptase may contribute to vascular permeability by the direct or indirect generation of bradykinin from kininogens [14]. Mast cell tryptase increases intracellular Ca^2+^, leading to elevation of paracellular permeability of colonocytes [15]. Intradermal injection of tryptase or mast cell secretagogue compound 48/80 in rats can induce the immediate cutaneous reaction and increase dermal microvascular permeability, which can be inhibited by potent and specific tryptase inhibitor nafamostat or synthetic tryptase inhibitor APC366 [16, 17]. However, the mechanism of enhancement of vascular permeability induced by tryptase is still not clear and need further study.

Vascular endothelial growth factor (VEGF), an endothelial cell mitogen that promotes angiogenesis, was initially identified as a vascular permeability factor (VPF) [18, 19]. VEGF interacts with two high-affinity tyrosine kinase receptors, VEGFR-1 (Flt-1) and VEGFR-2 (KDR/Flk-1), to increase microvascular permeability and induce angiogenesis [20]. In patients with delayed hypersensitivity, the amount of VEGF produced in lesional scales was approximately 25 times higher than that in normal stratum corneum [21]. In patients with allergic contact dermatitis, the mRNA levels of VPF/VEGF and two VPF/VEGF vascular endothelial cell receptors (Flt-1 and Flk-1) were all strikingly overexpressed in dermal microvascular cells [22]. Interestingly, tryptase, VEGF, and VEGF receptors all abundantly reside in the regions of dermal allergic and hypersensitive reactions. Based on the above reports, we hypothesized that tryptase may increase the dermal microvascular permeability as well as edema through regulating the expression of VEGF and VEGF receptors.

In the present study, we evaluated the effect of VEGF on hyperpermeability induced by purified human *β*-tryptase or human mast cell supernatant in cultured human dermal microvascular endothelial cells (HDMECs) and investigated the effect of tryptase and tryptase inhibitor (APC366) on VEGF and VEGF receptor (Flt-1, Flk-1) expression. The results provide the evidence that VEGF is involved in the increase of tryptase-induced microvascular permeability, which represents a novel pathway for controlling allergic reaction in skin.

## 2. Materials and Methods

Culture media, reagents, and SuperScript First-Strand Synthesis System for RT-PCR were purchased from Invitrogen (Carlsbad, CA, USA). SV Total RNA Isolation Kit was from Promega (Madison, WI, USA). SYBR Green real-time PCR Master Mix was from Toyobo Company (Osaka, JP). Primary antibody against von Willebrand factor (vWF), CD34, vascular endothelial growth factor (VEGF), fms-like tyrosine kinase (Flt-1), kinase insert domain containing receptor (Flk-1), and Glyceraldehydes-3-phosphate dehydrogenase (GAPDH) were purchased from SantaCruz Biotechnology, Inc. (Santa Cruz, CA). Antihuman VEGF Antibody for inhibiting VEGF was obtained from R&D Systems (Minneapolis, MN, USA). SuperSignal West Pico Chemiluminescent Substrate was obtained from Pierce Biotechnology, Inc. (Rockford, IL, USA). *β*-trypatase was kindly provided by Dr. Shunlin Ren (Division of Gastroenterology, Virginia Commonwealth University, Richmond,VA, USA). All other reagents were from Sigma-Aldrich Chemical Co. (St. Louis, MO) unless otherwise mentioned.

### 2.1. Isolation, Culture, and Identification of Human Dermal Microvascular Endothelial Cells (HDMECs)

The method of HDMECs isolation and culture was set up based on literatures published previously [23–26]. Briefly, human neonatal foreskins were cut into small pieces and digested by 0.5 mg/mL Dispase dissolved in sodium acetate at 37°C for 1 h. After removal of the epidermis, the dermal fragments were treated with 1% collagenase I at 37°C for 1 h. The microvascular segments were passed through a 100-*μ*m nylon mesh cell strainer, collected, and purified by Percoll gradient centrifugation. The fraction with a density <1.048 g/mL, which was rich in microvascular fragments, was removed and applied to gelatin-precoated tissue-culture dishes and cultured in Dulbecco's modified Eagle's medium (DMEM; 1000 mg/L glucose) supplemented with 10 mM HEPES, 10 mM L-glutamine, 15 U/mL heparin, 1 *μ*g/mL hydrocortisone acetate, 325 *μ*g/mL glutathione, 0.05 mM dibutyryl cyclic AMP, 5 *μ*g/mL insulin, 5 *μ*g/mL transferrin, 5 *μ*M 2-mercaptoethanol, 100 U/mL penicillin, 100 *μ*g/mL streptomycin, and 20% fatal bovine serum. The HDMECs were identified on the basis of morphological characteristics, immunofluorescent staining of von Willebrand factor (vWF) and CD34. All experiments used HDMECs at passages 2–4.

### 2.2. Culture of Human Mast Cell Line HMC-1

The human mast cell line HMC-1 was kindly obtained from Second Military Medical University, Shanghai, China. The cells were cultured in 75 cm^2^ flasks in Iscove's modified Dulbecco's medium (IMDM) supplemented with 10% fetal bovine serum (FBS), 100 IU/mL penicillin and 100 *μ*g/mL streptomycin in humidified air with 5% CO_2_ at 37°C. Collected HMC-1 cells were activated and degranulated in the addition of prodegranulating agent a23187. The HMC-1 supernatant containing tryptase is collected, centrifuged, filtered, and then used as conditioned medium (henceforth referred as HMC-1 supernatant) in the following experiments. The activity of tryptase released from HMC-1 was quantified by monitoring hydrolysis of tosyl-L-Gly-ProLysp-nitroanilide (t6140) using a standard spectrophotometric assay at 405 nm wavelength. Tryptase was released from HMC-1 cells in a23187 (a prodegranulating agent) dose-dependent manner and HMC-1 cells density-dependent manner (Supplemental Figure  2 available online at doi:10.5402/2012/941465). The optimal stimulation and release were achieved by incubating HMC-1 cells (1 × 10^7^/mL) for 2 h with a23187 (1 *μ*g/mL) at 37°C.

### 2.3. Determination of Vascular Permeability in Cultured HDMECs

As described in previous literature [27], HDMECs were grown to confluent monolayer on gelatin-coated membranes in double-chamber tissue culture plates (Transwell membrane, 0.4 *μ*M pore size, Corning Costar). After 48 h, chambers were examined microscopically for integrity and uniformity of endothelial monolayers. The confluent monolayers were incubated with APC366 (250 *μ*g/mL), anti-VEGF antibody (0.1 *μ*g/mL) or SU5614 (5 *μ*M) following its activation by either tryptase or HMC-1 supernatant for 18 h as described. At the end of the incubation period, FITC-conjugated dextran (1 mg/mL, Mr 42,000; Sigma-Aldrich) was added to the upper chambers, and fluorescence in the lower chamber was measured 1 h later with a fluorescence reader. Experiments were performed in triplicate and repeated 3 times.

### 2.4. Determination of Gene Expression of VEGF and Its Receptors

Total cell lysates of HDMECs were extracted on ice with 1% NP40, 0.5% sodium deoxycholate and 0.1% SDS in PBS with proteinase inhibitor cocktail (Sigma). Fifty *μ*g total proteins were loaded on 7.5% SDS-PAGE for detection of the specific proteins, including VEGF, and its receptors Flt-1 and Flk-1, using GAPDH as loading control. Western blot analysis was performed as previously described [28]. All Western blot experiments were repeated at least three times with separate cells preparation.

Total RNA was extracted using SV Total RNA Isolation Kit (Promega, Wisconsin, WI) according to the supplier's instructions. Two micrograms of total RNA were reversely transcribed and amplified. The relative mRNA levels were measured by real-time PCR as described previously [28]. Specific primer pairs for VEGF, Flt-1, Flk-1, and GAPDH were listed in Table 1.

### 2.5. Statistical Analysis

Data were presented as mean  ±  SEM. Statistical significance was assessed by one-way ANOVA and discrepancies between groups were considered statistically significant at *P* < 0.05.

## 3. Results

### 3.1. Culture and Identification of Human Dermal Microvascular Endothelial Cells (HDMECs)

All HDMECs gave typical confluent cobblestone appearance (Supplemental Figure  1(a)) and had positive reactions to the antibodies against vWF (Supplemental Figure  1(b)) and CD34 (Supplemental Figure  1(c)). Negative control without first antibody exhibited no staining (Supplemental Figure  1(d)). More than 90% cells were positive for vWF and CD34, suggesting the purity of the primary cells exceeded 90%.

### 3.2. Determination of the Tryptase Activity in HMC-1 Supernatant

To confirm the existence of tryptase in the conditioned medium, we incubated the HMC-1 supernatant with substrate (t6140, N-Tosylglycyl-L-prolyl-L-lysine 4-nitroanilide acetate salt, 8 mmol/L) in the presence and absence of prodegranulating agent a23187 (1 *μ*g/mL) for 10 minutes in the reaction buffer (40 mM HEPES, 0.12 M NaCl, pH 7.4). OD value of the reaction was detected by spectrophotometer at 405 nm each 30 seconds. As shown in Supplemental Figure  2(a), the change of OD405 (formation of t6140-derived product digested by tryptase) was linear for at least 10 minutes, and 5 minutes was chosen as the reaction time. Tryptase was released in the HMC-1 supernatant, which is increased dramatically by prodegranulating agent a23187 (Supplemental Figure 2(b)). a23187 stimulated HMC-1 cells to release tryptase dose-dependently (Supplemental Figure  2(c)). On the other way, tryptase was released from HMC-1 cells by 1 *μ*g/mL a23187 in cell density-dependent manner (Supplemental Figure  2(d)). In the following experiments, HMC-1 supernatant was prepared by using 1 × 10^7^ HMC-1 cells treated with 1 *μ*g/mL a23187.

### 3.3. Effect of Tryptase/HMC-1 Supernatant on the Permeability of HDMECs

**Figure 1:**
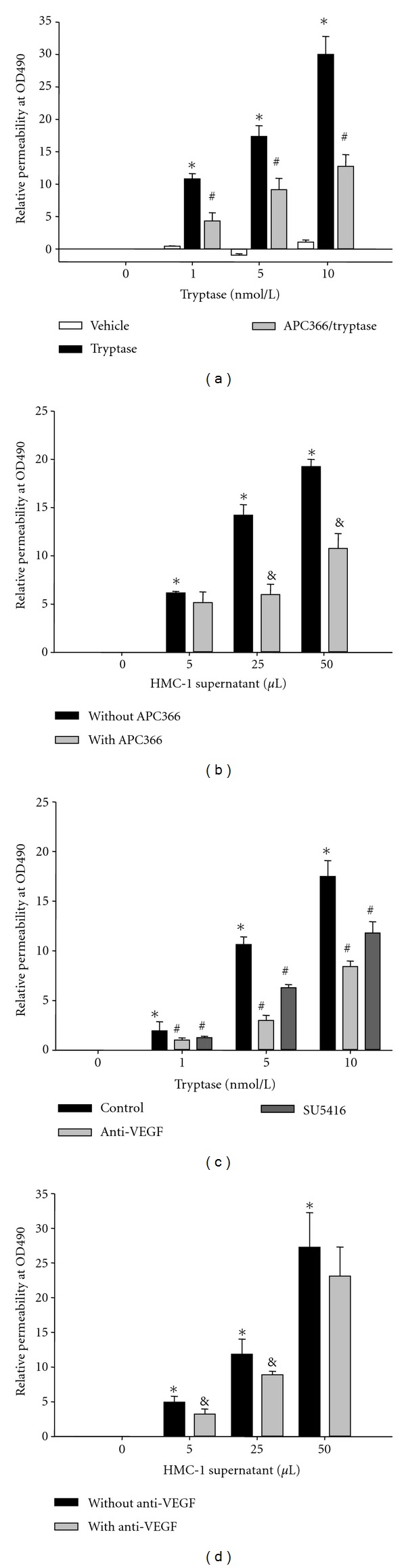
Effect of tryptase and HMC-1 supernatant on the permeability of HDMECs. As described in Methods, the permeability of HDMECs after indicated treatment was detected by measuring fluorescence in the lower chamber at 490 nm after incubation with FITC-dextran for 1 h in the upper chamber. The changes of OD490 in the lower chamber after 1 h of incubation were calculated for the permeability of HDMECs. (a) Effect of tryptase at different concentrations on the permeability of HDMECs in the presence or absence of APC366. The heparin was used as Vehicle control. (b) Effect of HMC-1 supernatant at different concentrations on the permeability of HDMECs with or without APC366. (c) Effect of anti-VEGF antibody and SU5416 on the increase of permeability stimulated by tryptase. (d) Effect of anti-VEGF antibody on the increase of permeability stimulated by HMC-1 supernatant. *P < 0.05 compared to the group of nonaddition. ^#^ *P* < 0.05 compared to the group only treated with tryptase. ^&^ *P* < 0.05 compared to the group only treated with HMC-1 supernatant.

As described in the method, the amount of FITC-dextran in the lower chamber leaked from the HDMECs layer was detected to measure the permeability of HDMECs. The permeability of HDMECs with different treatments was quantified by the percentage of OD490 change. The confluent monolayers were treated with tryptase or HMC-1 supernatant for 18 h in the presence or absence of APC366 (a selective inhibitor of tryptase, 250 *μ*g/mL) pretreatment. As shown in Figure 1(a), tryptase significantly increased the permeability of HDMECs in a dose-dependent manner, which was resisted by APC366. Because *β*-tryptase was added into HDMECs accompanied by heparin as stabilizer, heparin control was also studied. It turns out that addition of heparin to HDMECs had no effect on the permeability.  Figure 1(b) showed that HMC-1 supernatant enhanced the permeability of HDMECs dose-dependently, which was resisted by APC366. To investigate whether VEGF is involved in the hyperpermeability, anti-VEGF antibody (0.1 *μ*g/mL) was preincubated on HDMECs to block VEGF. The data was normalized to groups treated with normal goat IgG. As a result, inhibition of VEGF significantly attenuated tryptase-induced permeability (Figure 1(c)), but only modestly attenuated HMC-1 supernatant-induced permeability (Figure 1(d)). SU5614, 3-[(2,4-demethylpyrrol-5-yl)methylidene]-indolin-2-one, is a small synthetic inhibitor of the catalytic function of the VEGF receptor (VEGFR-2; Flk-1/KDR) tyrosine kinase. It was used to strengthen the evidence that VEGF is involved in the hypermeability caused by tryptase. As shown in Figure 1(c), pretreatment of 5 *μ*M SU5416 with HDMECs dramatically attenuated tryptase-induced hypermeability.

### 3.4. Effect of Tryptase on the VEGF, Flt-1, and Flk-1 Protein Levels in HDMECs

**Figure 2:**
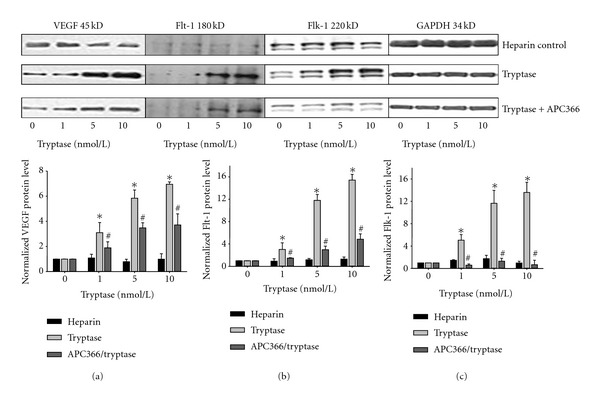
Effect of tryptase on the VEGF, Flt-1, and Flk-1 protein levels in HDMECs with or without APC366. HDMECs were treated with different concentrations of tryptase for 18 h in the absence or presence of APC366 (250 *μ*g/mL). The protein levels of VEGF (a), Flt-1 (b), and Flk-1 (c) were determined by Western blot and normalized to GAPDH. The heparin control was also analyzed. *P < 0.05 compared to the group of nonaddition. ^#^ *P* < 0.05 compared to the group only treated with tryptase at the same concentration.

To study the mechanism of resistance of tryptase-induced hyperpermeability by anti-VEGF antibody, the protein levels of VEGF, Flt-1, and Flk-1 in HDMECs of indicated treatments were analyzed by Western blot. Different concentrations of tryptase were added into HDMECs for 18 h in the absence or presence of APC366. The heparin control was also analyzed. As a result, addition of different concentration of tryptase to HDMECs in culture significantly increased the protein levels of VEGF (Figure 2(a)), Flt-1 (Figure 2(b)), and Flk-1 (Figure 2(c)), which was resisted by APC366, a synthetic tryptase inhibitor. However, there was no effect on these protein expressions following the treatment of heparin control.

### 3.5. Effect of Tryptase on the VEGF, Flt-1, and Flk-1 mRNA Levels in HDMECs

**Figure 3:**
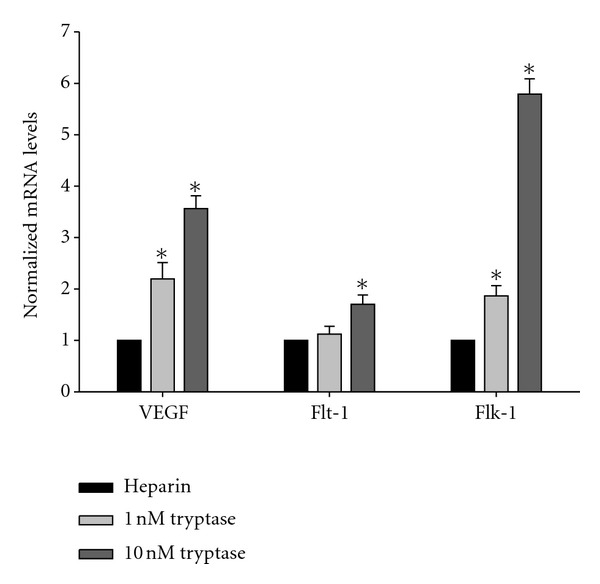
Effect of tryptase on the VEGF, Flt-1, and Flk-1 mRNA levels in HDMECs. Different concentrations of tryptase (0, 1, and 10 nmol/L) were added into HDMECs for 6 h. The mRNA levels of VEGF (a), Flt-1 (b), and Flk-1 (c) were determined by Real-time RT-PCR and normalized to GAPDH. The heparin control was also analyzed. *P < 0.05 compared to the group of heparin control.

To further study the mechanism of resistance of tryptase-induced hyperpermeability by anti-VEGF antibody, the effect of tryptase on VEGF, Flt-1, and Flk-1 expressions in HDMECs at mRNA level was analyzed by Real-time RT-PCR. GAPDH was determined in parallel and used as an internal standard. Different concentrations of tryptase were added into HDMECs for 6 h. The expression levels were normalized to heparin control. As shown in Figure 3, tryptase upregulated VEGF, Flt-1, and Flk-1 mRNA levels significantly.

## 4. Discussion

In the present study, we demonstrated that both mast cell tryptase and HMC-1 supernatant promote vascular hyperpermeability in cultured human dermal microvascular endothelial cells (HDMECs), which can be significantly blocked by anti-VEGF and SU5416 (inhibitor of VEGF receptor, VEGFR-2/Flk-1). Furthermore, tryptase increases the expression of VEGF and its receptors (Flt-1 and Flk-1), which can be inhibited by synthetic tryptase inhibitor (APC366). These results provide the evidence that VEGF is involved in the increase of tryptase-induced microvascular permeability, which represents a novel pathway for controlling allergic reaction in skin.

Tryptases are predominantly mast cell-specific serine proteases with pleiotropic biological activities [8, 29]. Under physiological conditions, tryptases are primarily detectable in mast cells and basophils and at least consist of *α*-tryptase and *β*-tryptase. *β*-tryptase appears to be the main isoenzyme that is expressed in human lung and skin mast cells, whereas in basophils *α*-tryptase predominates [30]. *β*-tryptase with physiological activities exists as tetrameric conformation, which is ionically bound to heparin proteoglycan [31]. Heparin stabilizes tryptase in its enzymatically active form [32, 33]. Therefore, heparin proteoglycan with identical concentrations was used as Vehicle control in our experiments.

Increasing evidences indicate that mast cell tryptase plays an important role in enhancement of vascular permeability [14–17]. Mast cell tryptase increases intracellular Ca^2+^, leading to elevation of paracellular permeability of colonocytes [15]. Mast cells control permeability of the intestinal epithelium by cleaving protease-activated receptor 2 (PAR_2_) on the basolateral membrane of colonocytes and activating extracellular signal-regulated kinases 1/2 (ERK1/2) [15, 17]. Our data also demonstrated that either purified tryptase or HMC-1-released tryptase stimulated vascular permeability in primary human dermal microvascular endothelial cells (HDMECs), which can be partly inhibited by synthetic tryptase inhibitor (APC366) (Figure 1). Tryptase inhibitors reduced but did not abolish the effects of HMC-1 mast cell supernatant on permeability of HDMECs, suggesting that mast cell mediators other than tryptase may also regulate vascular permeability.

In addition to tryptase, mast cells release a number of mediators that act directly on the vasculature to produce vasodilatation and increase permeability, including vascular endothelial growth factor (VEGF) [34]. VEGF is an endothelial cell-specific mitogenic peptide and plays a key role in vasculogenesis, angiogenesis, and stimulation of vascular permeability [35–38]. The VEGF family includes VEGF-A, placenta growth factor (PlGF), VEGF-B, VEGF-C, and VEGF-D. The original member of the VEGF family, VEGF-A, also known as vascular permeability factor, was characterized as a potent inducer of vascular permeability [19, 35, 39, 40]. In the present work, VEGF-A is represented as VEGF. VEGF binding to the 2 types III receptor tyrosine kinases VEGF receptor-1 (VEGFR-1/Flt-1) and VEGFR-2 (Flk-1) are primarily expressed in vascular endothelial cells. Previous studies have revealed that VEGF expressions by epidermal keratinocytes and endothelial expression of VEGF receptors are upregulated in cutaneous inflammation [21]. However, whether VEGF and its receptors are involved in the tryptase-induced hyperpermeability is unknown. Therefore, in the present study, we investigated the effect of tryptase on the expressions of VEGF and its receptors (Flt-1 and Flk-1). The results showed that tryptase significantly increased the mRNA and protein levels of VEGF and its receptors in HDMECs, which can be inhibited by APC366 (Figures 2 and 3). Furthermore, SU5614, a potent inhibitor of VEGF, and anti-VEGF effectively resisted the tryptase-induced hyperpermeability (Figure 1). Hereby, VEGF is at least partially responsible to the enhancement of permeability induced by tryptase. However, the specific mechanism of how tryptase stimulates expression of VEGF and its receptors should be elucidated in the further study.

In conclusion, mast cells tryptase significantly increased the expressions of VEGF and its receptors (Flt-1 and Flk-1) and promoted microvascular permeability in HDMECs, which can be reversed by VEGF inhibitor. The results indicated that VEGF is involved in the increase of dermal microvascular hyperpermeability by tryptase. These findings may lead to novel means of controlling allergic reaction in skin.

## Supplementary Material

All HDMECs gave typical confluent cobblestone appearance (Supplemental Figure 1(a)), and had positive reactions to the antibodies against vWF (Supplemental Figure 1(b)) and CD34 (Supplemental Figure 1(c)). Negative control without first antibody exhibited no staining (Supplemental Figure 1(d)). The expressions of vWF and CD34 were also quantified with flow cytometry. Exceed 90% cells were positive for vWF and CD34, which suggested the purity of the primary cells exceeded 90%.
Measurement of the tryptase activity of HMC-1 supernatant: To affirm the existence of tryptase in the conditioned medium, we incubated the HMC-1 supernatant made with HMC-1 suspension in the presence and absence of pro-degranulating agent a23187 (1 **µ**g/mL) with substrate (t6140, N-Tosylglycyl-L-prolyl-L-lysine 4-nitroanilide acetate salt, 8 mmol/L) for 10 minutes in the reaction buffer (40 mM HEPES0.12 M NaClpH 7.4). OD value of the reactions were detected by spectrophotometer at 405 nm each 30 seconds. The control is set with only the reaction buffer. As shown in Supplementary Figure 2(a), the change of OD405 (formation of t6140-derived product digested by tryptase) was linear for at least 10 minutes. Then 5 minutes was chosen to be the reaction time. Tryptase was released in the HMC-1 supernatant, which is increased dramatically by pro-degranulating agent a23187 (Supplementary Figure 2(b)). A23187 stimulated HMC-1 cells to release tryptase dose-dependently (Supplementary Figure 2(c)). On the other way, tryptase was released from HMC-1 cells by 1 **µ**g/mL a23187 in density-dependent manner (Supplementary Figure 2(d)). In the following experiments, HMC-1 supernatant was prepared using 1 × 107 HMC-1 cells/mL treated with 1 **µ**g/mL a23187.

## Figures and Tables

**Table 1:** Primer pairs used to amplify PCR products.

Gene	Sequence (5′-3′)	Product size	Annealing T (°C)	GeneBank no.
VEGF	Forward: CAACATCACCATGCAGATTATGC	132 bp	60°C	NM_001033756
	Reverse: CCCACAGGGATTTTCTTGTCTT			
Flt-1	Forward: TGGCTGCGACTCTCTTCTG	118 bp	60°C	NM_002019
	Reverse: CAAAGGAACTTCATCTGGGTCC			
Flk-1	Forward: GGCCCAATAATCAGAGTGGCA	104 bp	60°C	NM_002253
	Reverse: TGTCATTTCCGATCACTTTTGGA			
GAPDH	Forward: CATGAGAAGTATGACAACAGCCT	113 bp	60°C	NM_002046
	Reverse: AGTCCTTCCACGATACCAAAGT			
