# Supplementary material for: VEGF Is Involved in the Increase of Dermal Microvascular Permeability Induced by Tryptase
Source: ISRN Dermatol. 2012 May 15;2012:941465. doi: 10.5402/2012/941465 (PMC3361195; doi:10.5402/2012/941465)
Supplement: Supplementary file 1 — All HDMECs gave typical confluent cobblestone appearance (Supplemental Figure 1(a)), and had positive reactions to the antibodies against vWF (Supplemental Figure 1(b)) and CD34 (Supplemental Figure 1(c)). Negative control without first antibody exhibited no staining (Supplemental Figure 1(d)). The expressions of vWF and CD34 were also quantified with flow cytometry. Exceed 90% cells were positive for vWF and CD34, which suggested the purity of the primary cells exceeded 90%. Measurement of the tryptase activity of HMC-1 supernatant: To affirm the existence of tryptase in the conditioned medium, we incubated the HMC-1 supernatant made with HMC-1 suspension in the presence and absence of pro-degranulating agent a23187 (1 µg/mL) with substrate (t6140, N-Tosylglycyl-L-prolyl-L-lysine 4-nitroanilide acetate salt, 8 mmol/L) for 10 minutes in the reaction buffer (40 mM HEPES0.12 M NaClpH 7.4). OD value of the reactions were detected by spectrophotometer at 405 nm each 30 seconds. The control is set with only the reaction buffer. As shown in Supplementary Figure 2(a), the change of OD405 (formation of t6140-derived product digested by tryptase) was linear for at least 10 minutes. Then 5 minutes was chosen to be the reaction time. Tryptase was released in the HMC-1 supernatant, which is increased dramatically by pro-degranulating agent a23187 (Supplementary Figure 2(b)). A23187 stimulated HMC-1 cells to release tryptase dose-dependently (Supplementary Figure 2(c)). On the other way, tryptase was released from HMC-1 cells by 1 µg/mL a23187 in density-dependent manner (Supplementary Figure 2(d)). In the following experiments, HMC-1 supernatant was prepared using 1 × 10^7^ HMC-1 cells/mL treated with 1 µg/mL a23187. [file 941465.f1.pdf]

Supplemental Information

Supplemental Fig.1

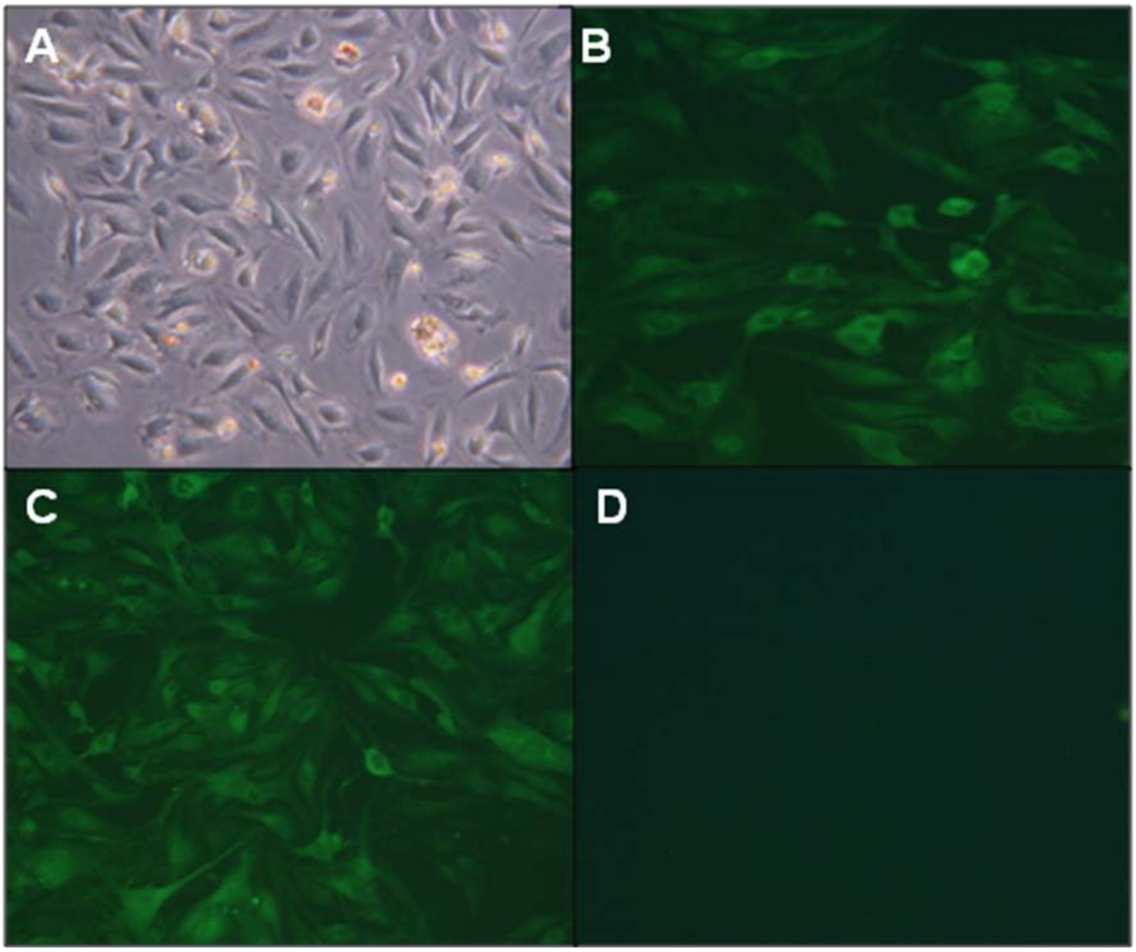

Supplemental Fig.1 Determination of purity of primary HDMECs by immunofluorescence. Morphology of passage 2 human dermal microvascular endothelial cells (HDMECs) was shown in (**A**). To confirm the purity of HDMECs, we used von Willebrand's factor (**B**) and CD34 (**C**) immunofluorescent staining, which are specific for endothelial cells. Negative control without first antibody staining was shown in (**D**). (X 200)

Supplemental Fig.2

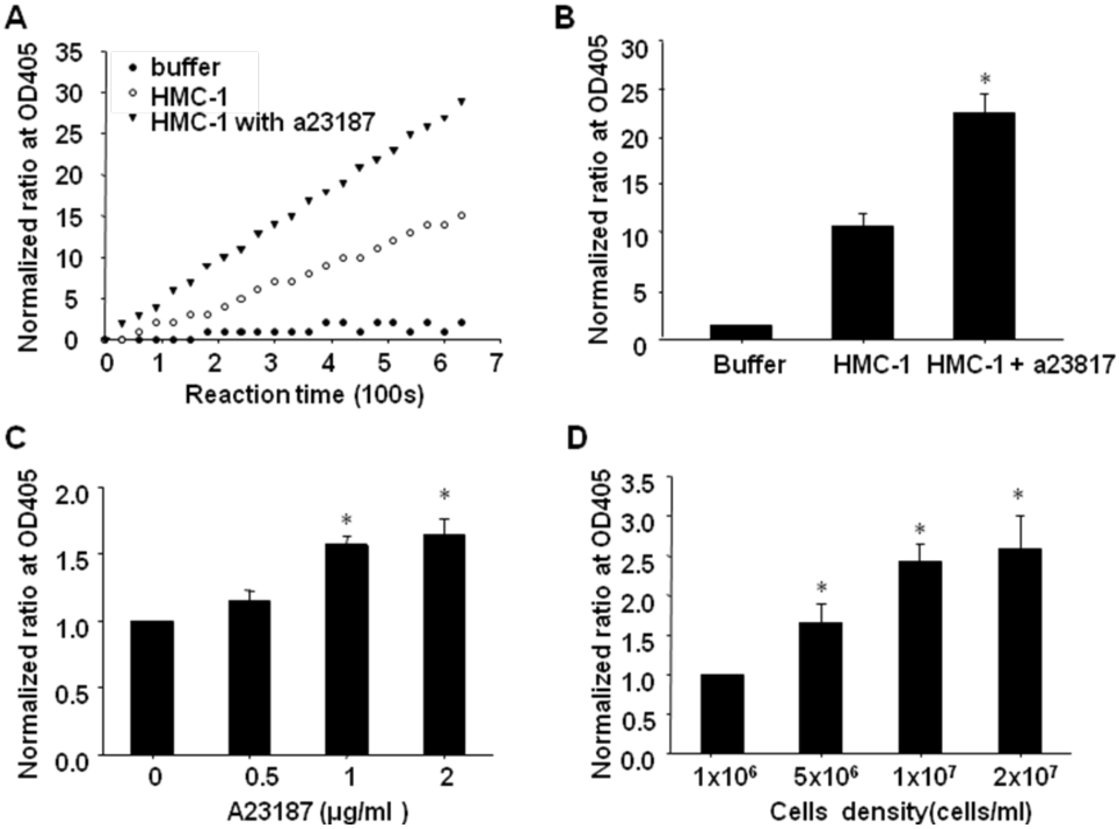

Supplemental Fig.2 Determination of the tryptase activity in HMC-1 supernatant. **A**: HMC-1 supernatant was incubated with substrate (t6140) in the presence or absence of a23187 for 10 minutes. OD value of the reactions was measured by spectrophotometer at 405nm each 30 seconds. The control is set with only the reaction buffer. **B**: Comparation of the tryptase activity of HMC-1 supernatant without and with pro-degranulating agent a23187. **C**: Tryptase activities of the HMC-1 supernatant stimulated by different concentrations of a23187. **D**: Tryptase activities of the HMC-1 supernatant with different cell densities, which were normalized to the group of 10^6 HMC-1 cells/ml. *P < 0.05 compared to control.
